# State and Evolution of the Investigación y Educación en Enfermería Journal from a Metric Analysis

**DOI:** 10.17533/udea.iee.v39n3e02

**Published:** 2021-11-05

**Authors:** Manuela Vélez Ramírez, Jaider Ochoa Gutiérrez, Marcela Suárez Tamayo

**Affiliations:** 1 Sistema de Bibliotecas. Universidad de Antioquia. Email: manuela.velezr@udea.edu.co Universidad de Antioquia Sistema de Bibliotecas Universidad de Antioquia Colombia manuela.velezr@udea.edu.co; 2 Grupo de Investigación Información, Conocimiento y Sociedad. Escuela Interamericana de Bibliotecología. Universidad de Antioquia. Email: jaider.ochoa@udea.edu.co Universidad de Antioquia Escuela Interamericana de Bibliotecología Universidad de Antioquia Colombia jaider.ochoa@udea.edu.co; 3 Grupo de Investigación Información, Conocimiento y Sociedad. Escuela Interamericana de Bibliotecología. Universidad de Antioquia Email: marsumayo@gmail.com Universidad de Antioquia Escuela Interamericana de Bibliotecología Universidad de Antioquia Colombia marsumayo@gmail.com

**Keywords:** bibliometrics, serial publications, scientific publication indicators., bibliometría, publicaciones seriadas, indicadores de producción científica., bibliometría, publicações seriadas, indicadores de produção científica.

## Abstract

**Objective:**

To analyze levels of production, reach, and thematic development of the *Investigación y Educación en Enfermería* journal from a scientometric analysis.

**Methods:**

The study collected 1,066 articles corresponding to the period between 1983 and 2020. The scientometric analysis was carried out from three components of descriptive analysis: performance of the publication, geographic reach, and thematic development. The first two used data consolidated from articles published in the *Open Journa*l*System*at Universidad de Antioquia. The third component captured the bibliographic references from the *Web of Science* and *Scopus* databases and from the *Google Scholar* and *Lens* academic search engines.

**Results:**

In terms of the production analysis, the Journal shows stable behavior sustained over time with international reach regarding authorship. In the thematic setting, the Journal concentrates on two large clusters: 1) research on human factors from different perspectives and 2) cross-sectional studies differentiated mainly by sex. With respect to emerging clusters, on one side, a thematic pillar is seen with studies in young adult population and another in matters related to the educational process and nursing students.

**Conclusion:**

The Journal has maintained outstanding behavior in terms of production over time, aligned with very good visibility for potential authors internationally; something not easily accomplished for most journals in Colombia. Likewise, its production has had a thematic domain to a greater extent related to human factors associated with the nursing practice.

## Resumen

**Objetivo:**

Analizar los niveles de producción, alcance y desarrollo temático de la revista *Investigación y Educación en Enfermería*a partir de un análisis cienciométrico.

**Métodos:**

Se recopilaron 1066 artículos correspondientes al periodo 1983-2020. El análisis cienciométrico se desarrolló a partir de tres componentes de análisis descriptivo: desempeño de la publicación, alcance geográfico y desarrollo temático. Para los dos primeros se utilizaron los datos consolidados de los artículos publicados en el Sistema *Open Journal System*de la Universidad de Antioquia. Para el último, se capturaron las referencias bibliográficas de las bases de datos *Web of Science*y*Scopus*, y de los buscadores académicos *Google Scholary* y *Lens*.

**Resultados:**

En términos del análisis de la producción, la Revista muestra un comportamiento estable y sostenido en el tiempo con un alcance internacional en cuanto a la autoría. En el ámbito temático, se concentra en dos grandes clústeres: 1) la investigación sobre factores humanos desde diferentes perspectivas y, 2) estudios transversales diferenciados principalmente por sexo. En cuanto a los clústeres emergentes, por un lado, se ve un pilar temático con estudios en población adulta joven y otro en asuntos relacionados con el proceso educativo y los estudiantes de enfermería.

**Conclusión:**

La Revista ha mantenido un comportamiento destacado en términos de producción en el tiempo, alineado con muy buena visibilidad para potenciales autores en el contexto internacional, lo que no es logro fácil para la mayoría de las revistas en Colombia. Asimismo, su producción ha tenido un dominio temático en mayor medida relacionado con los factores humanos asociados a la práctica de la enfermería.

## Resumo

**Objetivo:**

Analisar os níveis de produção, abrangência e desenvolvimento temático do periódico Nursing Research and Education a partir de uma análise cienciométrica.

**Métodos:**

Foram coletados 1.066 artigos correspondentes ao período de 1983-2020. A análise cienciométrica foi desenvolvida a partir de três componentes da análise descritiva: desempenho da publicação, abrangência geográfica e desenvolvimento temático. Para os dois primeiros, foram utilizados os dados consolidados dos artigos publicados no *Open Journal System* da Universidade de Antioquia. Para este último, as referências bibliográficas foram capturadas nas bases de dados *Web of Science* e *Scopus*, e nos motores de busca acadêmicos *Google Scholar* e *Lens.*

**Resultados:**

Em termos de análise da produção, a Revista mostra um comportamento estável e sustentado ao longo do tempo com abrangência internacional em termos de autoria. Na área temática, a Revista concentra-se em dois grandes grupos: 1) pesquisa sobre fatores humanos a partir de diferentes perspectivas e, 2) estudos transversais diferenciados principalmente por sexo. No que diz respeito aos clusters emergentes, por um lado, existe um pilar temático com estudos na população adulta jovem e outro nas questões relacionadas com o processo educativo e discentes de enfermagem.

**Conclusão:**

A Revista tem mantido um desempenho notável em termos de produção ao longo do tempo, alinhado com uma visibilidade muito boa para potenciais autores a nível internacional; algo que não é fácil de conseguir para a maioria das revistas na Colômbia. Da mesma forma, sua produção teve um domínio temático em maior medida relacionado aos fatores humanos associados à prática de enfermagem.

## Introduction

Within the environment of scientific communication, scientific journals constitute a means *par excellence* for the dissemination of knowledge product of research activity.(1) This means permits increasing the knowledge base of the fields and academic communities, becoming essential to devise mechanisms that facilitate measuring or evaluating their performance.(2) Metric analysis of production in scientific journals permits identifying signs in their behavior that can best guide their management.(3) Commonly, bibliometric and scientometric studies have been oriented by analyzing the impact factor as indicator *par excellence* for journals. However, with the rise of digitality and positioning of open access, these types of indicators have been heavily questioned and,(4,5) hence, new metrics are proposed that permit recognizing in greater detail the performance of the journals and a tool to strengthen editorial management.(4)

This article sought to analyze the production levels, reach, and thematic development of the *Investigación y Educación en Enfermería* (IEE) journal from a scientometric analysis.(6) This Journal is a scientific publication of the Faculty of Nursing at Universidad de Antioquia, focusing on support, development, and visibility of the Nursing discipline. It has a periodicity of three numbers per year and is indexed in the main information sources, like *Web of Science*, *Scopus* and *PubMed Central*. The observation window concentrates on analyzing the production, its reach, most-cited articles, and the thematic domain the publication has had. This required analysis of 1 066 articles corresponding to the journal’s history, specifically, to the period comprised between 1983 and 2020.

This called for the consolidation of the database of articles from the Journal, which is available in the *Open Journal System* software managed by the University. To analyze the thematic domain, data of articles indexed in *Web of Science*, *Scopus*, Lens and *Google Scholar* was used. The results presented permit elucidating a general state of the Journal; seeing the behavior of the production over time, by authors, institutions, and countries; identifying articles with the best citation indexes; besides providing a panorama of the themes of greater relevance that have been worked in the articles published. This seeks to be a first systematization effort that offers a panorama based on data to support strengthening editorial management processes.

Overall, the Journal shows stable behavior sustained over time with international reach regarding authorship. In the thematic setting, this publication concentrates on two large clusters of themes corresponding, initially, to research on human factors and then to cross-sectional studies to analyze risk factors, health status or behavior differentiated by sex. The Journal has maintained outstanding behavior in terms of production over time and with very good visibility for potential authors internationally; something not easily accomplished for most journals in the country. Likewise, its production has had a thematic domain to a greater extent related to human factors associated with the nursing practice.

## Methods

This study reviews 1,066 articles from the *IEE* journal, since the publication of its first number in 1983 until June 2021. The aim was to see the patterns of publication, collaboration by authors, geographic reach, and thematic development from the scientometric analysis(1-3,6,7) and social network analysis.(8-11) Recovery of articles was conducted through the Open Journal System (OJS) at Universidad de Antioquia. After obtaining a preliminary list, revision was made of each of the metadata, seeking to eliminate repeated registries and identifying missing information.

The data systematized from each of the articles were title, full name of each author, institutional affiliation of each, country of origin of each institution, key words, and year of publication. The OJS yielded automatically all the data, except for the institutional affiliation of each of the authors and country of origin; these were filled out manually. Upon obtaining all the data, the work proceeded with standardization, emphasizing on the authors, institutions, and countries.

Regarding information on the citation and thematic relation, the data were captured from *Web of Science*, *Scopus*, Lens, and *Google Scholar*. This permitted obtaining a general panorama of the state of the Journal in scientific information sources. To analyze and visualize the data, the *Power BI* and *Tableau* applications were used; for the visualization of networks of co-authorship and co-occurrence of words, *VOS Viewer* was used.

Analysis instruments. This study answers three fundamental questions: (i) *what has been the performance in terms of productivity of the IEE journal since its first publication until now?* This journal has been in uninterrupted publication for almost 40 years, which speaks for itself of a hard-won solidity. With systematized data of the number of articles, number of authors and institutions, year-to-year, its evolution will be demonstrated. This type of information permits the Journal’s directives to review significant changes in editorial policies and subsequent analysis of how these have affected the production of articles and their impact. (ii) *What has been the Journal’s geographic reach regarding scientific production and how have the dynamics of collaboration between authors and institutions come about?* The capture of institutional affiliations and countries of origin of each of the authors permit observing the origin of the articles published and the collaboration dynamics among authors and, hence, institutions. (iii) *What are the main thematic axes the Journal has had?* By using the methodology of social network analysis and the *VOSViewer* software*,*(8) the construction of word co-occurrence analysis networks is carried out; that is, identify the joint occurrences of two terms in a text to determine the thematic domain.(11) This specific case used the Lens indexing base, with > 900 articles, which captures the terms under the MeSH organization scheme, of great use being that it is a controlled vocabulary focused on health sciences. Networks permit visualizing the clusters or thematic blocks that permit recognizing the Journal’s domain.

## Results

The following presents the principal’s descriptive results of the IEE journal. Initially, activity or production indicators are described, then the reach and co-authorship, and - lastly - the thematic analysis.

### What has been the behavior of the Journal’s publication of articles over time?

As observed in Graphic 1, the Journal has had stable behavior in recent years in the production of articles. It is striking that at no time has publication been stopped and, therefore, the Journal’s positioning has not been affected. During the last decade, growth in publications is notable due to the increase of a third number per year. However, although there is a decrease in the number of articles published in the last four years, this does not indicate difficulties in the editorial management process; on the contrary, it may be related with the reduction of articles per number for greater quality control and, thereby, a higher rejection rate for manuscripts received. This could be interpreted in terms of maturity and exigency of the editorial policies.

**Graphic 1:**
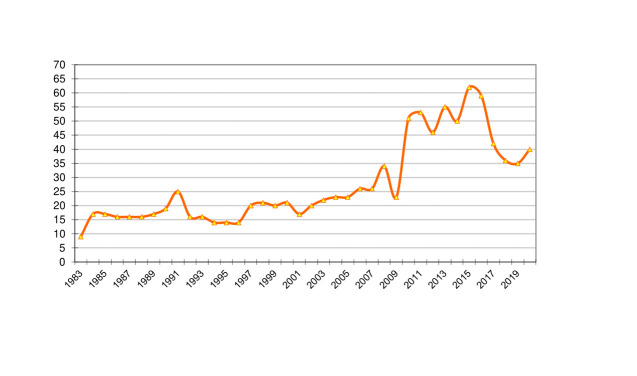
Frequency of articles published from 1983 to 2020 in the IEE Journal

### What are the principal institutional affiliations of the authors who published in the Journal?

Table 1 shows the 18 most-frequent institutions related with the authors who publish in the Journal, with the first being Universidad de Antioquia, an expected condition, considering its shared origin. However, it is striking that, in order of production, international institutions appear above others from the national order. It can be seen in the case of Brazilian universities, like Universidade Federal do Rio Grande do Sul, with 94 articles and Universidade de São Paulo, with 92 articles. Likewise, other institutions, like Shiraz University of Medical Sciences, from Iran with 73 affiliations; and, lastly, Pontificia Universidad Católica in Chile with 72 affiliations identified. This could be interpreted in terms of the reach and the interest the Journal generates internationally and not only at the local and national levels. 

**Table 1:** Frequency of articles by institutional affiliation of the authors

Institution	Frequency
Universidad de Antioquia	723
Universidade Federal do Rio Grande do Sul	94
Universidade de São Paulo	94
Schiraz University of Medical Science	73
Pontificia Universidad Católica de Chile	72
Universidad Nacional de Colombia	64
Universidad Autónoma de Nueva León	37
Universidade Federal de Ceará	35
Universidade Estadual Paulista	34
Universidade Federal de Santa Catarina	34
Universidad del Valle	49
Universidade Federal São João del Rei	29
Universidad Federal do Rio Grande do Sul	28
Universidad de Cartagena	24
Universidade Federal de Paraná	24
Universidade do Estado do Rio de Janeiro	24
Universidade Estadual de Maringá	23

### What are the principal nationalities related with the authors who publish in the Journal?

**Figure 1:**
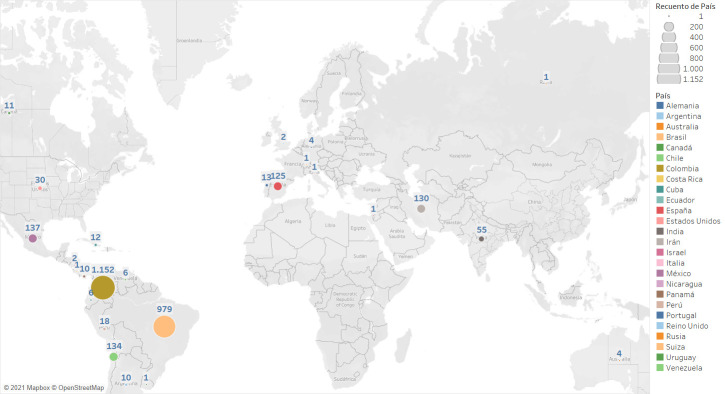
Frequency of the undersigning authors of the articles according to country

In relation to the authors’ origins, as shown in Figure 1, most publications have been from authors of Colombian nationality, followed by Brazilians, Mexicans, and Chileans; this could be translated into good regional visibility at Latin American level. Nevertheless, also striking is the number of authors from the European block, mainly Spain and Portugal; and from the Asian continent, especially from Iran and India. These data could be read as greater reach of visibility and interest in the Journal’s publication, which agrees with the analysis of institutional affiliation.

### What are the principal themes addressed in the Journal?

The thematic analysis by co-occurrence of words permits seeing the relations and closeness among terms worked in the Journal’s articles. This specific case used MeSH terms, which are typical of the specialized language of the health field and have a standardization process. In this case, this type of analysis of social networks provides a panorama of the Journal’s thematic domain, identifying which are the concepts most worked and how those relations are perceived as thematic clusters. This, on the matter of editorial management, permits the Journal to analyze its thematic strengths and, from there, guide policies, seek and motivate authors on the theme, and plan numbers of greater degree of specialization.

**Figure 2:**
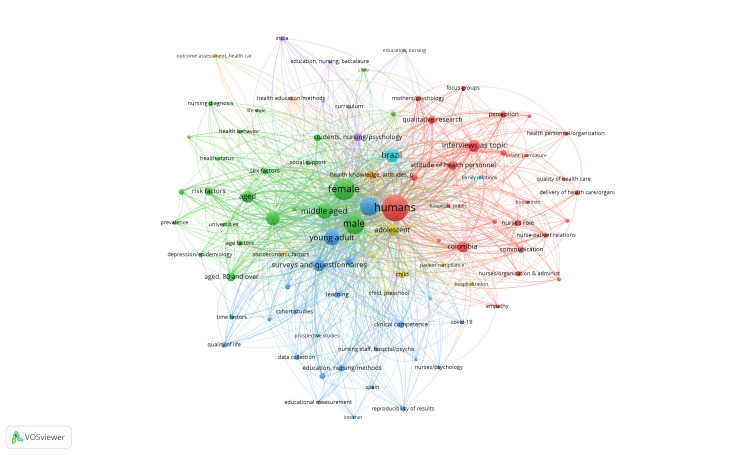
Principal themes developed in the Journal’s articles

In general, the Journal concentrates on two large thematic clusters (Figure 2) and another two emerging or complementary. A first cluster related with studies of human factors from different perspectives and the second corresponding to cross-sectional studies mainly differentiated by sex. Regarding the emerging clusters, on one side, thematic pillar is seen related with studies on young adult population and on the other, on issues related with the educational process and nursing students. The following analyzes each thematic cluster.

Specifically, the thematic cluster can be seen, corresponding to the term Humans; the size of the node shows the importance and recurrence of this concept in the Journal’s publications. The term presents a co-occurrence link with terms, such as qualitative research and analysis techniques associated with this type of research; this supposes that an interesting number of articles use this type of methodology. The relationship can also be seen of this concept with studies on the profile and role of nurses, showing permanent concern of the field for this topic of analysis. Evidence exists of studies that, within this framework, work the patient-nurse relation, communication, and some values with relation to humanism. In transversal manner, it could be understood that multiple related studies have focused on Colombia, which is consistent with the origin of the largest number of authors the Journal has had.

The second most relevant cluster shows a thematic development related to cross-sectional studies associated with gender factors. According to other terms associated, these studies could focus on analyzing risk factors, health status or in men and women. Likewise, a node of interest is shown related to the concept of adult. This term can be linked to others, such as young adults, surveys, and questionnaires; this could be associated with multiple studies that focus on applying these instruments to the knowledge of health factors in this population. In turn, other relationships can be seen with quality of life, psychological factors, and family. In addition, the relationship between terms associated with COVID-19 studies and educational resources is also noteworthy. From this cluster, a small emerging cluster can be seen referring to the terms adolescents, children, and hospitalization. This could be interpreted with relation to the term adult, such as the existence of articles that analyze by age groups.

Lastly, an emerging cluster may be seen with a principal node related with the term nursing students. Upon seeing the relations available, interesting terms appear, like education and curriculum, a trend that could be taken as interest of the publications for the educational process of nurses and for the lives of university students. Network analysis of co-occurrence of words permitted identifying the Journal’s thematic dimension, seeing which themes are of greater focus or interest and, therefore, an analysis framework that allows promoting editorial strategies to support the consolidation of topics. This exercise is shown as an interesting field of work to continue potentiating from the Journal’s direction and, in that sense, make decisions that support enhancing the editorial management.

## Discussion

This section is structured by thinking more on taking advantage of the data results, to provide some ideas to keep in mind when managing the Journal, hence, what will be seen ahead focuses on taking some of those elements to make proposals. It is important, from the start, to recognize the trajectory of the IEE Journal, which can not only be seen as a reference in its field for the University but also for the country and the region. It is significant to consider this, bearing in mind the difficulties endured by many journals for their sustainability over time and, above all, for their indexing in important sources, like *Scopus*, *Web of Science* and *PubMed Central*.

The production behavior shows a very stable Journal, with a production that has been maintained over time, not only to boost research from the University but, also, from multiple latitudes. Analyses demonstrate sustained production, principally by authors from Universidad de Antioquia, but much attention is drawn to international visibility where it is possible to see researchers from Brazil, Chile, Mexico, Spain, Portugal, Iran, and India. Considering the foregoing, it is imperative to continue thinking of diverse visibility strategies in different channels, continue motivating researchers from multiple places to publish and evaluate works, participate actively in scientific networks and networks of journals; sustain presence in the main indexing systems and diversify ways of communicating the publication.

In addition, it is suggested for the Journal to design digital marketing strategies that allows it to gain greater presence in social networks, in general networks, like *Facebook*, *Twitter, and Linked-in,* as well as in specialized networks, like *Research Gate*. This implies, for example, insisting on the design of new ways of communicating the publication and characterizing the audiences that may be interested in the contents of the Journal.

Within the thematic setting, the results evidence a variety of important themes for publication. It is important to continue conducting these types of exercises, considering the possibility of promoting numbers focused on thematic pillars. Likewise, being able to identify authors with those domains to motivate them to publish; in addition to crossing the thematic analysis with identifying or characterizing the citation, given that it could account for which themes are stronger and of greater interest for the academic community. In turn, it is worth to relate with co-authorship analysis to identify the Journal's momentum in building a scientific community.

Digital curation is necessary or constant revision of the metadata available in the OJS. When executing this project, problems emerged with the normalization of authors and data on institutional affiliation; for this, it is essential to work hand in hand with the Journals System team at Universidad de Antioquia to, thus, constantly verify the quality of the information. It is also important to monitor indexing systems, given that problems could exist with the information indexed therein or, even, loss of visibility and citations could occur.

These types of exercises support, under the focus of data and evidence management, the strengthening of editorial management. In this sense, it is important to continue with a line of work that supports performing new analyses and constructing metrics. Due to such, future exploration is proposed of the type of citation, that is, characterize the types of citations the Journal has; this will permit knowing which can be the greatest strength in terms of impact, like, for example, if the Journal is taken as reference for formative processes, to support new research, or for other types of documents that require the base it offers.

Lastly, just as it has insisted on designing strategies for visibility in social networks, it is also important to boost the monitoring and development of metrics in these channels, given that, although evidence exists in the literature that shows the importance of these types of indicators for visibility and citation, each publication has its peculiarities to keep in mind to enhance said processes. In this sense, if Altmetric indicators are systematically designed (12-14) for the Journal, this could help to define new strategies that help the publication’s that support the publication’s editorial management.
